# 

**Published:** 1890-08

**Authors:** 


10 cts. a copy. AUGUST, 1890. $1.00 per year.

### HALL'S JOURNAL OF HEALTH

ESTABLISHED 1854

**Headquarters—218 to 222 Fulton St., near Greenwich. Office, Room 18.**

Entered as Second-Class Matter at the Post-Office, New York.

